# SGLT2 Inhibitor-Induced Sympathoexcitation in White Adipose Tissue: A Novel Mechanism for Beiging

**DOI:** 10.3390/biomedicines8110514

**Published:** 2020-11-18

**Authors:** Jennifer R. Matthews, Lakshini Y. Herat, Aaron L. Magno, Shelley Gorman, Markus P. Schlaich, Vance B. Matthews

**Affiliations:** 1 Dobney Hypertension Centre, School of Biomedical Science—Royal Perth Hospital Unit, University of Western Australia, Crawley, WA 6009, Australia; jen.matthews@uwa.edu.au (J.R.M.); lakshini.weerasekera@uwa.edu.au (L.Y.H.); 2 Research Centre, Royal Perth Hospital, Perth, WA 6000, Australia; aaron.magno@uwa.edu.au; 3 Telethon Kids Institute, University of Western Australia, Perth, WA 6009, Australia; shelley.gorman@telethonkids.org.au; 4 Dobney Hypertension Centre, School of Medicine—Royal Perth Hospital Unit, University of Western Australia, Crawley, WA 6009, Australia; markus.schlaich@uwa.edu.au; 5 Department of Cardiology and Department of Nephrology, Royal Perth Hospital, Perth, WA 6000, Australia

**Keywords:** sympathetic nervous system, sodium glucose cotransporter 2 inhibition, Dapagliflozin, beiging, adipose tissue, hypertension

## Abstract

Recent preclinical data show that sodium glucose cotransporter 2 (SGLT2) inhibitors are able to reduce weight gain and induce beiging in white adipose tissue (WAT). We have previously shown that in neurogenic hypertensive Schlager (BPH/2J) mice, treatment with the SGLT2 inhibitor, Dapagliflozin, reduced blood pressure and prevented weight gain. Here we show that chemical sympathetic denervation achieved by systemic administration of 6-hydroxy-dopamine (6-OHDA) reduces body weight and the heightened sympathetic nervous system (SNS) innervation in WAT. Furthermore, we demonstrate that 2 weeks of Dapagliflozin treatment increases SNS innervation in WAT of hypertensive mice. This increase is accompanied by a non-significant elevation in mRNA levels of the *Ucp1* and *Pgc-1α* genes, which are markers of beiging. No significant difference in the mRNA levels of the inflammatory mediators *Il-6* and *Tnf-α* were detected in WAT of Dapagliflozin treated mice. These findings suggest that SGLT-2 inhibitor-associated prevention of weight gain may be mediated, at least in part, by inducing the beiging of WAT.

## 1. Introduction

Over 600 million adults and over 100 million children are estimated to be obese worldwide with the global prevalence of obesity predicted to continue to rise [1]. Obesity is a driving force in the development of several metabolic disorders including cardiovascular disease, diabetes and hypertension [2]. As obesity is a result of an imbalance between energy intake and expenditure, a new therapeutic strategy that can restore the energy balance is required.

Recent research into the role of adipose tissue in regulating the energy balance through either fat storage or heat expenditure has made it an attractive target for therapies aimed at reducing obesity [3]. Historically, adipose tissue has been categorized into two distinct groups. There is white adipose tissue (WAT), which is primarily involved in the storage of energy, and the more energetically active brown adipose tissue (BAT), which is involved in energy expenditure [4]. BAT can be stimulated to increase energy expenditure as required [5]. Historically, BAT was considered a means by which human infants responded to cold through thermogenesis. It was believed that in human adults, the presence of BAT was only vestigial and not involved in normal physiology [6]. However, advances in imaging technology, specifically positron emission tomography-computerized tomography (PET-CT) with ^18^F-fluorodeoxyglucose (^18^F-FDG) as a tracer, allowed for several large scale studies to demonstrate functionally active BAT in adult humans [7,8,9]. There is now evidence for a third type of adipose tissue, beige adipose tissue, which has been found in WAT depots but is more energetically active like BAT [3]. The induction of beige adipocyte biogenesis or beiging in WAT depots and the activation of BAT is an attractive strategy to combat obesity [10].

Cold exposure and exercise are methods by which BAT activation and beiging can be induced in humans [10]. A range of dietary compounds, including capsaicin, resveratrol, curcumin, green tea catechins, berberine, omega-3 polyunsaturated fatty acids and all-trans retinoic acid, have been examined in animals and humans as promoters of BAT thermogenesis and beiging with mixed results [4]. Similarly, various pharmaceuticals such as catecholamines, thiazolidinediones, fenofibrate and FGF21 mimetics have been trialled to stimulate BAT activation and beiging but have generally been found to exert unwanted adverse effects [3,4]. There has been recent evidence that both BAT thermogenesis and beiging are dependent on the sympathetic nervous system (SNS), with both brown and beige adipose tissue being highly sympathetically innervated [11,12]. Cao et al., have demonstrated that the direct microinjection of the specific SNS neurotoxin, 6-hydroxydopamine (6-OHDA) into the BAT of C57Bl/6 mice causes SNS denervation in the BAT but increases SNS outflow to WAT with subsequent increases in both tyrosine hydroxylase and UCP1 protein expression, resulting in enhanced beiging [13]. However, denervation of WAT by 6-OHDA microinjection abolished the upregulation of tyrosine hydroxylase and UCP1 expression. Our group has been examining the impact of sodium glucose cotransporter 2 (SGLT2) inhibitors on SNS activity in various models. We have previously shown that administering the SGLT2 inhibitor, Dapagliflozin, to either C57Bl/6 mice or hypertensive BPH/2J mice on a high fat diet reduces SNS innervation and activation in the kidneys and the heart, in addition to reducing weight gain [14,15].

Here we present novel data on the disparate effects of 6-OHDA and Dapagliflozin on SNS activity in the WAT of a mouse model characterized by genetically determined sympatho-excitation. We also provide for the first-time evidence of Dapagliflozin-induced beiging of WAT which is associated with non-significant elevations in the expression of the *Ucp1* and *Pgc-1α* mRNA

## 2. Experimental Section

### 2.1. Animals

Experiments were conducted in 12-week-old male and female Schlager mice (BPH/2J strain) rederived and bred at the Animal Resource Centre (ARC, Perth, Australia). All animal experimentation was carried out at the Royal Perth Hospital animal holding facility in accordance with the guidelines of the Royal Perth Hospital Animal Ethics Committee (R537/17-20; approved: 15/08/17). Mice were acclimatized for 1 week, and then all mice were fed a high fat diet (Specialty Feeds, Glen Forrest, Australia) for the 2 weeks of treatment. All mice were given unrestricted access to water and food. After acclimatization, mice were administered 5 intraperitoneal injections of 100-mg/kg 6-OHDA (Sigma, St. Louis, MO, USA) in 0.9% saline [16] over a 2-week period. In a separate series of experiments, we explored the effect of SGLT2 inhibition on sympathetic activity in vivo using BPH/2J mice fed a high fat diet for 2 weeks and mice received either vehicle or 40-mg/kg Dapagliflozin (4C Pharma Scientific Inc., Guelph, ON, Canada) via oral gavage every 2 days for 2 weeks. Body weights were measured weekly for all mice. Fasting blood glucose was measured using the Accu-Chek Performa blood glucose monitoring system (Roche Diagnostics, North Ryde, Australia). Urine glucose levels were measured for all mice using the Keta-Diabur-Test 5000 (Roche Diagnostics). At the conclusion of the experiment, all mice were anesthetized with methoxyflurane and were euthanized by cervical dislocation. Gonadal WAT was collected and either fixed in paraformaldehyde and subsequently embedded in paraffin wax, or snap-frozen in liquid nitrogen and stored at −80 °C [14].

### 2.2. Immunohistochemistry

WAT was sectioned at 5 μm onto silane-treated microscope slides and de-waxed in xylene and rehydrated in ethanol. Antigen retrieval was performed on the slides by heating in EDTA buffer (pH 8.5; Sigma-Aldrich). Slides were treated with 3% hydrogen peroxide and then blocked in 5% FCS in PBS/0.1% Tween-20. Tyrosine hydroxylase was detected with rabbit anti-tyrosine hydroxylase (AB152; Merck Millipore, Bayswater, Victoria, Australia). Antibody binding was detected with anti-rabbit secondary antibodies conjugated to HRP, followed by treatment with DAB. Tissues were counterstained with haematoxylin before being dehydrated in ethanol and cleared in xylene and mounted with DPX (Sigma-Aldrich). Photomicrographs were taken of stained WAT from mice using a Nikon Eclipse Ti Microscope (Nikon Instruments Inc., Amsterdam, The Netherlands). Tyrosine hydroxylase positive nerves were counted in random fields of view [15].

### 2.3. Enzyme-Linked Immunosorbent Assays

Frozen WAT was homogenized and analyzed for norepinephrine content using the mouse norepinephrine NA ELISA kit (CSB-E07870m) (Cusabio, Wuhan, China) according to the manufacturer’s instructions [15].

### 2.4. Real-Time Measurement of mRNA

Total RNA was prepared using TRIzol Reagent (Invitrogen, Carlsbad, CA, USA) and Quantitect Primer Assays (Qiagen, Melbourne, Australia) were used for detection of *Pgc-1a*, *Ucp1*, interleukin-6 (*Il-6*) and tumour necrosis factor-α (*Tnf-α*) mRNA with internal primers for detection of *Eef1α*, a house-keeping gene [17].

### 2.5. Statistical Analyses

All quantitative data are presented as mean ± SEM. Significance was determined using Student’s t test. A *p* value < 0.05 was considered statistically significant. Graphs were generated using GraphPad Prism 7 (GraphPad Software, San Diego, CA, USA) and the analyses were performed using Microsoft Excel (Microsoft Corporation, Redmond, WA, USA).

## 3. Results

### 3.1. Chemical Denervation with 6-OHDA Significantly Reduces Body Weight and Sympathetic Nervous System (SNS) Innervation in White Adipose Tissue (WAT)

**Figure 1:**
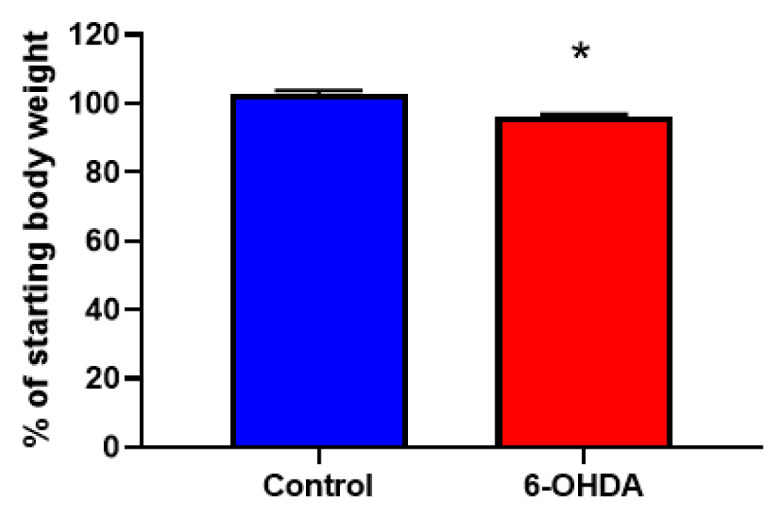
Chemical denervation with 6-hydroxydopamine (6-OHDA) significantly reduced body weight in Blood Pressure High/2J (BPH/2J) mice, *n* = 4 mice/group. * *p* < 0.002; all data presented as mean ± standard error of the mean (SEM).

**Figure 2:**
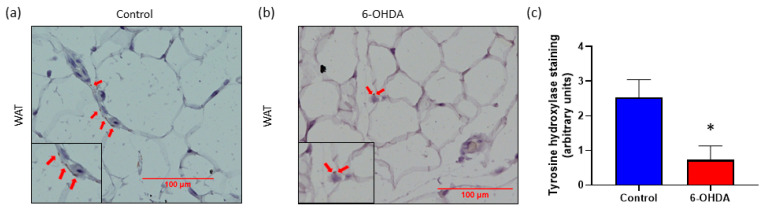
Chemical denervation with 6-OHDA significantly reduces sympathetic nervous system (SNS) innervation in white adipose tissue (WAT). (**a**,**b**) Representative immunohistochemistry images of tyrosine hydroxylase expression in WAT. Tyrosine hydroxylase (TH) staining is brown in color and is indicated with arrows. Insets indicate close-ups of TH staining. Size bar = 100 µm. Magnification 200×. (**c**) Quantitation of tyrosine hydroxylase expression in WAT from BPH/2J mice, *n* = 3–5 mice/group. * *p* < 0.05; all data are presented as mean ± SEM.

In our current study, we used a neurogenic hypertensive mouse model, known as the Schlager (BPH/2J) mouse which has heightened sympathetic tone. We have previously shown in this strain that use of the chemical denervation agent, 6-OHDA, significantly reduces hypertension [14]. Using 6-OHDA, we were able to reduce body weight (Figure 1) and SNS innervation in BPH/2J mice as evidenced by a decrease in tyrosine hydroxylase staining in WAT (Figure 2). As tyrosine hydroxylase is a reliable marker of sympathetic innervation, we showed that sympathetic hyperactivity can be dampened in our BPH/2J mice with chemical denervation techniques.

### 3.2. Sodium Glucose Cotransporter 2 (SGLT2) Inhibition Promotes Glucosuria in Blood Pressure High/2J (BPH/2J) Mice

**Figure 3:**
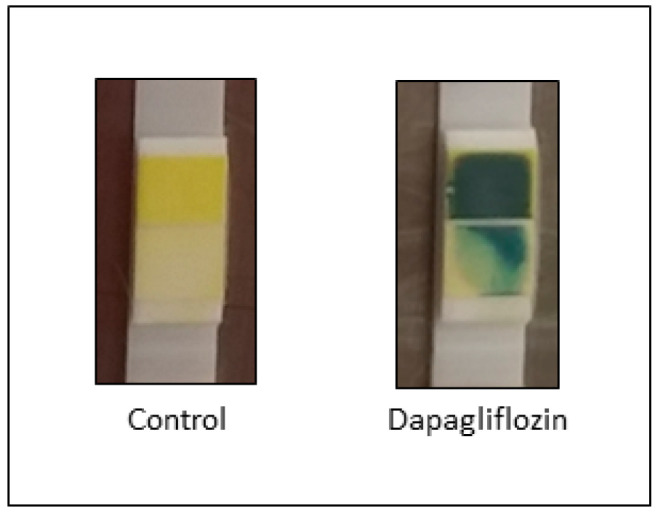
Representative image of glucose levels in urine after 11 days of Sodium glucose cotransporter 2 (SGLT2) inhibition. Dapagliflozin was administered at a dose of 40 mg/kg. Yellow = 0 mmol/L glucose and blue = 278 mmol/L glucose.

BPH/2J mice receiving Dapagliflozin via oral gavage for 11 days exhibited glucosuria (Figure 3), in comparison to the control mice receiving vehicle.

### 3.3. SGLT2 Inhibition Promotes Increased SNS Innervation in WAT

**Figure 4:**
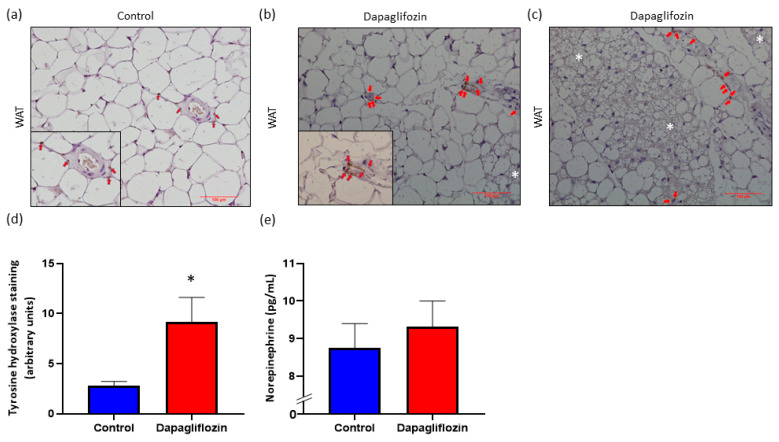
SGLT2 inhibition promotes increased SNS innervation in WAT. (**a**–**c**) Representative immunohistochemistry images of tyrosine hydroxylase expression in WAT. Tyrosine hydroxylase staining is brown in color and is indicated with arrows. Insets indicate close-ups of TH staining. Size bar = 100 μm. Magnification 200×. Brown adipose tissue like morphology is indicated with a white asterisk. (**d**) Quantitation of tyrosine hydroxylase expression in WAT from BPH/2J mice; *n* = 9–11 mice/group. * *p* = 0.011. (**e**) Norepinephrine in WAT from BPH/2J mice; *n* = 7–8 mice/group. All data are presented as mean ± SEM.

In our previous studies [14,15], we demonstrated that SGLT2 inhibition reduces SNS activation in organs, such as the kidneys and the heart, and reduces hypertension. We next sought to assess the impact that SGLT2 inhibition has on SNS innervation and activity within WAT from BPH/2J mice treated with Dapagliflozin. Interestingly, SGLT2 inhibition significantly increased tyrosine hydroxylase staining compared with vehicle-treated control mice (Figure 4). In addition, we examined the levels of norepinephrine, the major neurotransmitter of the SNS, in the adipose tissue and found that Dapaglifozin treatment elevated norepinephrine levels (Figure 4e). These observations strongly suggest that SGLT2 inhibition is associated with sympatho-excitation in WAT of this mouse model.

### 3.4. Inhibition of SGLT2 Promotes Elevation of Markers of Brown Adipose Tissue (BAT) in WAT

**Figure 5:**
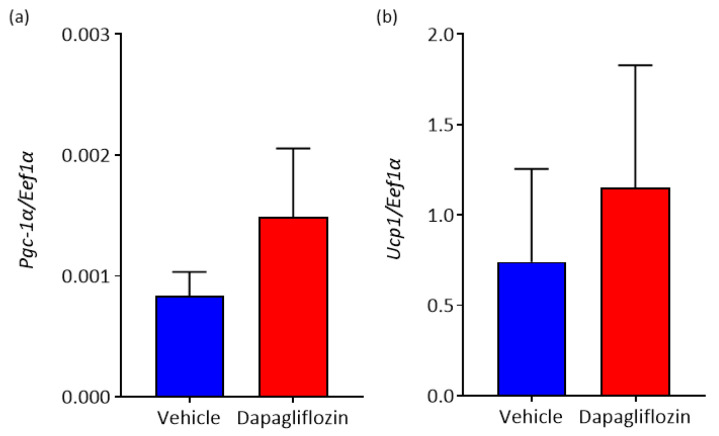
Inhibition of SGLT2 promotes elevation of markers of BAT in WAT. (**a**) *Pgc-1a* mRNA expression and (**b**) *Ucp1* mRNA expression; *n* = 7–8 mice/group. All data are presented as mean ± SEM.

Beige adipocytes are present in WAT and once stimulated, convert to a brown fat, leading to an increase in thermogenesis. There are many transcription factors involved in beiging, one of which is *Ucp1* [18]. Most pharmacological approaches used to induce beiging are successful by inducing *Pgc-1α*. We hypothesized that SGLT2 inhibition would increase expression of both *Ucp1* and *Pgc-1α*. Indeed, mRNA analysis revealed that SGLT2 inhibition non-significantly elevated expression of both genes in WAT (Figure 5).

### 3.5. The Effect of SGLT2 Inhibition on Adipokine Expression in WAT

**Figure 6:**
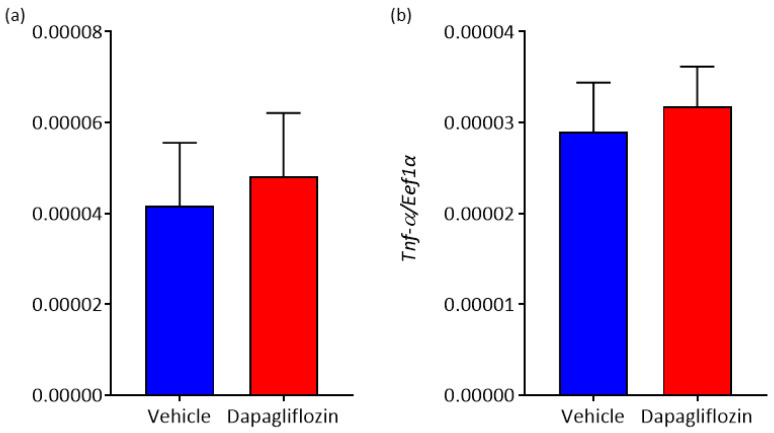
The effect of SGLT2 inhibition on pro-inflammatory cytokine expression in WAT. (**a**) Interleukin (*Il-6*) mRNA expression and (**b**) *Tnf-α* mRNA expression; *n* = 7–8 mice/group. All data are presented as mean ± SEM.

Finally, we sought to determine if SGLT2 inhibition could modulate expression of adipokines such as *Il-6* and *Tnf-α*. *Il-6* has been known to increase sympathetic activity [19] and *Tnf-α* is a well-known pro-inflammatory cytokine. SGLT2 inhibition was found to have no effect on either adipokine in our study (Figure 6).

## 4. Discussion

In this study, we continue to investigate the impact of pharmaceutical agents on the SNS in a mouse model of hypertension and the downstream ramifications of these treatments. We have previously shown that in hypertensive BPH/2J mice, both 6-OHDA and Dapagliflozin are able to reduce SNS activity in the heart and kidney and prevent increases in blood pressure [14]. Importantly, we also demonstrated that BPH/2J mice on a high fat diet receiving Dapagliflozin gained significantly less weight than their control counterparts [14]. In preclinical experiments, the SGLT2 inhibitors, Empagliflozin and Canagliflozin have reduced weight gain in mouse models of obesity [20,21]. SGLT2 inhibitors have also consistently been shown to promote weight loss in randomized clinical trials [22]. In addition, SGLT2 inhibitors also promote cardiovascular benefits [23,24]. Here we sought to determine if the lower weight gain associated with Dapagliflozin treatment in hypertensive mice was at least partially due to the regulation of the SNS and through beiging in WAT.

We first demonstrated that the systemic administration of the SNS denervation agent, 6-OHDA, to BPH/2J mice significantly reduces the heightened SNS innervation in WAT, as evidenced by the reduction of tyrosine hydroxylase expression. This supports the observations of Cao et al., demonstrating decreased SNS activity in WAT following direct injection of 6-OHDA into WAT [13]. Interestingly, in BPH/2J mice receiving Dapagliflozin, there was significantly increased tyrosine hydroxylase expression in WAT (Figure 4d) suggesting elevated SNS activity as opposed to the decreased SNS activity observed with 6-OHDA treatment. However, it should be noted that the tyrosine hydroxylase positivity we observed in WAT was less than we had previously seen in either kidneys or the heart. This increased SNS activity in the WAT of BPH/2J mice receiving Dapagliflozin is also in contrast to the decrease in SNS activity that we previously observed in other organs, specifically, the kidneys and the heart [14]. Therefore, the effects of SGLT2 inhibitors are tissue-dependent [23,24]. A trend of higher norepinephrine levels in the WAT of BPH/2J mice receiving Dapagliflozin was also observed which supports the finding of increased SNS activity in WAT (Figure 4e).

In our previous study, we demonstrated that Dapagliflozin treatment significantly retarded weight gain after 2 weeks of therapy [14]. Interestingly, the administration of 6-OHDA resulted in weight loss in our current study (Figure 1). However, it is complicated to compare the weight loss effects of Dapagliflozin and 6-OHDA. This is because Dapagliflozin promotes numerous metabolic benefits of which sympatho-inhibition is only one mechanism in kidney and heart. Conversely, sympatho-excitation occurs in the WAT after Dapagliflozin treatment in our current study. It is important to reiterate that regulation of the SNS by SGLT2 inhibitors is only one beneficial mechanism, which could impact weight loss. On the other hand, 6-OHDA directly promotes whole body sympatho-inhibition and is associated weight loss in our mouse model.

It is known that there are beneficial effects associated with the activation of neurons. In the context of the parasympathetic nervous system, its activation acts to dampen the SNS and subsequently dull the “fight or flight” response. Recently, Dapagliflozin has been found to regulate cardiovascular activity by increasing the activity of the parasympathetic nervous system in C57Bl/6 mice [25]. It is well known that the increased activity of the parasympathetic nervous system is associated with increased tyrosine hydroxylase expression [26,27].

During the assessment of tyrosine hydroxylase expression levels in WAT after SGLT2 inhibition, cells with BAT-like morphology were identified (Figure 4b,c). As increased SNS activity has been reported as one of the mechanisms of beiging [13], we propose that Dapagliflozin treatment induced “early” beiging in WAT of BPH/2J mice. In support of this, non-significant elevated mRNA levels of the BAT-selective gene *Ucp1* and the upstream mediator of *Ucp1*, *Pgc-1α*, were detected in WAT of BPH/2J mice receiving Dapagliflozin. Increased expression levels of PGC-1α and UCP1 have also been detected in mouse models of obesity that have been treated with the SGLT2 inhibitors Canagliflozin [20] and Empagliflozin [21], respectively. Wei et al., found that administering 60 mg/kg of Canagliflozin to C57Bl/6 mice on a high fat diet for 14 weeks reduced the rate of their weight gain as we have seen in our hypertensive mice [20]. Xu et al., showed that the long term (16 weeks) administration of 10 mg/kg of Empagliflozin to C57Bl/6 on a high fat diet also reduces weight gain [21]. Interestingly, significant reductions in weight gain and increased UCP1 expression were only observed at the higher Empagliflozin concentration (10 mg/kg) and not at the lower 3 mg/kg Empagliflozin concentration [21]. This suggests that the effects of SGLT2 inhibitors may be dose dependent. By increasing the dosage administered to our hypertensive mouse model to see if it would amplify the positive effects, we have observed an interesting avenue of further investigation. As mice in both of the aforementioned obesity studies [20,21] were treated with SGLT2 inhibitors for a longer time than the BPH/2J mice were in our study, we hypothesize that extending our time course of treatment may potentially also enhance the beneficial effects of Dapagliflozin.

Further variation between the SGLT2 inhibitors capacities to induce beiging in WAT could be related to their pharmacokinetics. For example, Empagliflozin has the greatest selectivity for human SGLT2 over SGLT1 (>2500-fold), compared to Dapagliflozin (>1200-fold) and Canagliflozin (>250-fold) [28]. A recent study using primary adipocytes has shown that SGLT2 inhibitors induce beiging via the AMPK signaling pathway [29]. Interestingly, Canagliflozin has been shown to be the most potent activator of the AMPK signaling pathway in a kidney cell line compared to both Dapagliflozin and Empaglflozin [30]. Further studies are required to determine if this hierarchy of potency also applies in adipose tissue.

Our data with Dapagliflozin significantly add to the literature in that SGLT2 inhibition can cause beiging in WAT. However, there are other possible benefits that SGLT2 inhibitors can provide beyond beiging including BAT activation and the regulation of inflammation. Xu et al., demonstrated by immunohistochemistry that Empagliflozin treatment decreased lipid accumulation in BAT and increased the number of UCP1 positive cells [21]. This suggests that the systemic administration of SGLT2 inhibitors not only stimulates beige adipocyte biogenesis in WAT but also induces BAT activation. PET-CT is the current gold standard for assessing BAT activity [31] and has been used to show acute β3 agonist-induced BAT activation in mice [32] and humans [32]. Future studies should be conducted using PET-CT to determine if SGLT2 inhibitors are able to acutely activate BAT and if there is any difference in potency between the inhibitors in both preclinical models and in humans. As adipose tissue is a major source of inflammatory mediators [33] we examined the impact of Dapagliflozin treatment on their expression in WAT from BPH/2J mice. We found no significant difference in the mRNA levels of interleukin-6 (*Il-6*) and tumour necrosis factor-α (*Tnf-α*) after 2 weeks of Dapagliflozin treatment. Increasing the length of Dapagliflozin treatment may impact *Tnf-α* and *Il-6* expression. Xu et al., showed that their 16 weeks of SGLT2 inhibitor treatment resulted in a significant decrease in *Tnf-α* and an increase in interleukin-10 (*Il-10*) mRNA levels in WAT [21]. Interestingly, we have previously observed a trend towards reduced TNF-*α* expression in the kidney of C57Bl/6 mice after receiving Dapagliflozin for 10 days [15]. We have also previously demonstrated an increase in IL-10 protein levels in both the heart and kidney of BPH/2J mice after 2 weeks of Dapagliflozin treatment [14]. IL-10 may play a significant role in the beiging of WAT as *Fas* mutant mice, which exhibit a significantly leaner phenotype and express elevated IL-10, show elevated levels of beiging in response to cold exposure compared to wild-type mice [34]. Future studies should involve a wider exploration of the expression of adipokines in WAT of Dapagliflozin treated mice. This includes the adipokine, leptin, which has been shown to have reduced mRNA levels in WAT following Empagliflozin treatment by Xu et al. [21]. In C57Bl/6 and Kimba mice, we have demonstrated lower serum levels of leptin following 8 weeks of Dapagliflozin treatment [35].

Although SGLT2 inhibitors were initially perceived to be a drug for the treatment of diabetes and its complications, it is now considered to also be beneficial for non-diabetic patients. Studies showing the benefits of SGLT2 inhibition in preclinical non-diabetic models [23,24] have led to randomized clinical trials exploring the benefits of SGLT2 inhibitor treatment in populations of non-diabetic patients [36,37].

## 5. Conclusions

In our study, Dapagliflozin promotes sympatho-excitation in WAT and we present histological evidence that is suggestive of beiging of WAT. Both findings might play a potential mechanistic role in the beneficial clinical effects of SGLT2 inhibition. However, the present data do not allow for the postulation of a causal relationship. Further research is required to determine the efficacy of SGLT2 inhibitors as therapeutic agents to combat obesity.
